# Pregnant or recently pregnant opioid users: contraception decisions, perceptions and preferences

**DOI:** 10.1186/s40834-018-0056-y

**Published:** 2018-03-27

**Authors:** Rebecca L. Fischbein, Bethany G. Lanese, Lynn Falletta, Kelsey Hamilton, Jennifer A. King, Deric R. Kenne

**Affiliations:** 1 Department of Family and Community Medicine, Northeast Ohio Medical University, 4209 St. Rt. 44, PO Box 95, Rootstown, OH 44272 USA; 2 College of Public Health, Department of Health Policy and Management, Kent State University, 750 Hilltop Drive, 322 Lowry Hall, P.O. Box 5190, Kent, OH 44242 USA; 3 Old Brooklyn Community Development Corporation, Cleveland, OH USA

**Keywords:** Contraception, Pregnancy, Opioid Drug Use

## Abstract

**Background:**

Multiple factors are linked to extremely high unintended pregnancy rates among women who use opioids, including various barriers to contraception adherence. These include patient level barriers such as lack of knowledge and education about highly effective contraception, and potential provider barriers. Using a mixed-methods framework to examine the contraception-related perceptions and preferences of opioid using women is a necessary next step to understanding this phenomenon.

**Methods:**

A mixed-method study was conducted which included both self-report questionnaires along with a semi-structured qualitative interview of opioid-using pregnant or recently pregnant women in two drug treatment facilities in Ohio.

**Results:**

Forty-two women completed the study. The majority of recent (75%) and total pregnancies were unintended. Male condoms were reported as the highest form of lifetime contraception used within the present sample (69%). Participants reported low lifetime use of long acting reversible contraception (LARC) (ranging from 5 to 12%). Participants preferred hormonal injections first (40%), followed by IUDs (17%). Reasons for preferences of injections and LARC were similar: not needing to remember, side effects, and long-term effectiveness.

**Conclusions:**

Most of the study population participants stated they would utilize contraception, particularly Tier 1 LARC methods, if freely available; however, high rates of unintended pregnancy were observed in this sample. This indicates the need for contraception education, and addressing the procedural, logistical and economic barriers that may be preventing the use of LARC among this population.

## Background

Women who misuse or abuse opioids are at great risk for unintended pregnancy with nearly eight out of every ten pregnancies unintended [1, 2]. Drug use during pregnancy can cause complications and potential adverse outcomes, including pre-term delivery, low-birth weight, microcephaly, and miscarriage [3]. Further, studies show that pregnant women with a substance use disorder (SUD) are less inclined to seek prenatal care, and often have higher rates of HIV, hepatitis, and other STIs [4]. Use of opioids during pregnancy and the number of newborns delivered with Neonatal Abstinence Syndrome (NAS) have been increasing rapidly in the United States [5]. Newborns suffering from NAS require special medical attention and other services [6] and are likely at-risk for future developmental delays, disabilities, and chronic medical conditions [7].

Increasing the use of contraception could reduce unintended pregnancy and associated health care problems among drug-using populations. While many of the available methods of contraception are effective when used correctly and consistently, in typical use among this population [8–11], pregnancy rates are much higher than for the general public, except for intrauterine devices (IUDs) and subdermal implants. In part, in real-world settings, this may be due to reduced adherence relative to non-drug using populations [12, 13]. Women in drug-using settings may face challenges and barriers that magnify their likelihood of unintended pregnancy [12, 14]. Because they have very low failure rates in both perfect and typical use, IUDs and subdermal implants are categorized as Tier 1 (defined as “very effective”) by the World Health Organization [15]. These long acting reversible contraceptive (LARC) methods are proven to be safe, effective, cost-effective, high in patient satisfaction and convenient (e.g., no daily or weekly requirements) [16–20]. Recently, LARC usage rates have increased in the United States with approximately 11% of contraception using women choosing LARC methods [17]. By improving contraception adherence, it is possible LARC methods also reduce the likelihood of unintended pregnancy among opioid using populations. However, while these products are safe and effective [20], there may be a gap in patient knowledge about LARC methods and/or patient and provider barriers to accessing LARC methods that may limit use among women in this population [21–23]. Most fundamentally, it is not known what women who use opioids think about these contraception options.

Women who use opioids may comprise a population who feel targeted to use these effective methods of contraception [2]. Investigating perceptions regarding contraception methods is crucial to ensure that effective methods are available for women to use in accordance with their preferences rather than because they feel pressured or coerced. However, decisions, preferences and perceptions regarding contraceptive methods are understudied among this complex group [24]. Previous research with this population strongly supports the inclusion of a qualitative component to better understand and eventually address their reproductive healthcare needs [25]. To date, only a few studies have examined the issue using qualitative methodology [26–28]. Semi-structured interviews conducted with seven women at a methadone clinic in rural New South Wales found that the participants were aware of contraception methods, but tended to use less effective methods [26]. In Australia, a qualitative study among 90 current or former injection drug-using women found that the reasons for contraceptive use differed among women. These participants also revealed varying concerns and barriers to different contraception methods [27]. One California study of substance-abusing pregnant women found that participants held misperceptions such as the belief that they were unable to become pregnant while using drugs due to the amenorrhea that is often associated with substance abuse [28].

To our knowledge, no mixed methods study has been conducted that examined decisions, preferences and perceptions related to contraceptive methods among opioid-using pregnant or recently pregnant women in the United States with a mixed methods approach. Given the dramatic increase in opioid use, including opioid use during pregnancy in the United States, this complex topic requires further investigation. Consequently, we asked pregnant or recently pregnant women who misuse or abuse opioids to share their thoughts on these topics.

## Methods

A comprehensive mixed-method study was conducted which included both self-report and interviewer-guided questions along with a semi-structured qualitative interview. The project was conducted in two stages with stage 1 interviews serving as a pilot study. Questions were exploratory and derived from the literature. Stage 2 expanded and replicated stage 1 with minor adjustments to the questions based on the evolution of the literature and newly emerging drug trends.

Interviews were conducted at two treatment facilities in Ohio. Treatment location 1 provided only non-residential services and treatment location 2 provided both residential and non-residential patient services. Using convenience sampling, participants were identified as eligible by program staff at the facilities. Participants were then recruited for the study by research staff. Individuals were eligible to participate if they met the following criteria: 1) currently or recently (within past 12 months) pregnant including miscarried or terminated pregnancies, 2) current or recent (within past 12 months) abuse/dependence of pharmaceutical opioids, 3) not actively psychotic, 4) English-speaking, 5) no current or past diagnosis of schizophrenia, 6) not currently suicidal, 7) not intellectually disabled, and 8) at least 18 years of age. Participants received $10 compensation for their participation. The final sample size reflects all women who agreed to participate and completed the study.

Stage 1 interviews were conducted in-person while stage 2 interviews were conducted remotely. The interviews were recorded with a digital recorder and each lasted approximately 90 min. A full board human subjects review was conducted due to the participants’ complex status. The participant population was identified as having multiple layers of vulnerability due to their status as pregnant, with substance use disorders, and further some may have had status as “prisoners” due to court-ordered residential treatment. The IRB approved this study and all protections were in place to protect this vulnerable population in this minimal risk study. Informed consent was obtained from all individual participants included in the study**.** The project was approved by the Kent State University institutional review board under protocol numbers: 12–277 and 13–063.

The questionnaires and semi-structured interview included the following domains: participant characteristics, pregnancy history, contraceptive utilization history, preferences and opinions, and perceptions of contraception effectiveness and dangers/side effects.

### Participant characteristics

Demographic questions reported in the current research include: age, race and marital status. Additional questions included lifetime use of means-tested programs including unemployment, cash assistance, food stamps, Medicaid and Woman, Infants and Children (WIC).

### Pregnancy history

Pregnancy history included questions about number of pregnancies, pregnancy outcome, whether each pregnancy was intended, and custody status for delivered pregnancies.

### Sexual history

Participants were asked about sexual history including the number of partners in the past year and whether they had ever traded sex for money, food, or drugs. Participants were also asked to indicate their STI history.

### Contraception use

Participants from treatment location 1 were asked an open-ended question “What kind(s) of birth control do you or have you used?” and participants from treatment location 2 were asked “What kind(s) of birth control do you currently use or have you used in the past?” The options for participants at Site 2 included: “the pill”, diaphragm, Vaginal Ring (NuvaRing), IUD, condom (male), female condom, the patch, sterilization/tubes tied, implant/Norplant, family planning (rhythm/pulling out), spermicides, Depo-Provera injection (3-month shot), Plan B/emergency contraceptives. These two questions were combined to represent contraception type lifetime use. Participants were asked about contraception access, including whether they had difficulty obtaining or purchasing contraception and how birth control was obtained. Participants were also asked decisions regarding contraception use, including reasons for contraception use and whether they primarily used contraception to prevent pregnancy, sexually transmitted infections (STI), or both. Women in treatment location 1 were asked about the situations in which they would or would not use condoms, while women in treatment location 2 were asked about the situations in which they would or would not use birth control.

### Contraception types and preferences

Questions about contraception type included what method they preferred to use, and if participants had access to free contraception 1) if they would use it, and 2) what method they would prefer and why.

### Perceptions of contraception effectiveness and dangers/side effects

Participants recruited from treatment location 2 were asked additional questions about their perceptions of effectiveness and dangers/side effects of different contraceptive methods. Participants were asked to respond to the following question “What percentage (or number of women out of 100) do you think would get pregnant in a year?” Possible responses included: < 1, 1–5, 6–10, > 10%, I don’t know. Participants were then asked to report “How bothered are you or would you be by the dangers or side effects of [contraception method]”. Possible responses included: not at all, a little, a lot, extremely, or I don’t know.

Data were analyzed using quantitative and qualitative methods. Quantitative data analyses included descriptive statistics which were conducted using SPSS Version 21. Qualitative data were coded for themes.

## Results

Table 1 reports participant characteristics. Forty-two participants provided complete information during the two-year study period 2012–2014; four individuals who enrolled did not and were not included in the analysis. Participants were predominantly white (81%) and single (74%), with an average age of 27.6 years. In addition, most women reported lifetime use of means-tested programs. The majority of participants indicated use of food stamps (88%), Medicaid (72%) and WIC (69%).

**Table 1:** Participant Characteristics (*n* = 42)

Variable	Statistic
*Age, Mean (SD)*	27.6 (5.14)
*Race*	
African American	4 (9.5%)
Caucasian	34 (81.0%)
Multi-racial	3 (7.1%)
Missing	1 (2.4%)
*Marital Status*	
Divorced/Separated	5 (11.9%)
Married	5 (11.9%)
Single	31 (73.8%)
Missing	1 (2.4%)
*Lifetime Use of Means-tests Programs*	
Woman Infants and Children (WIC)	29 (69.0%)
Medicaid	31 (72.1%)
Unemployment	2 (4.8%)
Food Stamps	37 (88.1%)
Cash Assistance	10 (23.8%)
*Treatment Location*	
Site 1	17 (40.5%)
Site 2	25 (59.5%)

### Pregnancy history

The participants reported a total of 137 pregnancies in the lifetime, of which 24 were ongoing (see Table 2). Subjects reported having custody of slightly less than half (*n* = 31) of the 74 children who had been delivered. Overall 75% of the 137 pregnancies were unintended. The average number of pregnancies was 3.3 per individual, ranging from one to nine.

**Table 2:** Pregnancy History

Variable	Statistic
*Total Pregnancies*	137
*Pregnancy Outcomes (n = 137)*	
Terminated	15 (11.0%)
Miscarried	23 (16.8%)
Delivered	74 (54.0%)
Currently Pregnant	24 (17.5%)
Missing	1 (0.7%)
*Pregnancies Per Individual, Mean (Range)*	3.3 (1,9)
*Custody of Delivered Children (n = 74)*	
Has Custody	31 (41.9%)
Does not have Custody	43 (58.1%)
*Intended Status of All Pregnancies (n = 137)*	
Intended	18 (13.1%)
Unintended	103 (75.2%)
Missing	16 (11.6%)
*Intended Status of Current/Recent Pregnancy (n = 42)*	
Intended	5 (11.9%)
Unintended	31 (73.8%)
Missing	6 (14.3%)

### Sexual history

The majority of participants (*n* = 25, 60%) indicated they had two or fewer sexual partners in the past year (see Table 3). Twenty-one participants (50%) reported having ever traded sex for money, drugs, or food. Nearly 62% (*n* = 26) of participants reported ever having been diagnosed with an STI, with chlamydia (*n* = 15, 36%) as the most frequently occurring.

**Table 3:** Sexual History

Variable	Statistic
*Number of Sexual Partners in Past Year*	
1	15 (35.7%)
2	10 (23.8%)
3	2 (4.8%)
4	3 (7.1%)
5	4 (9.5%)
6–10	2 (4.8%)
Over 50	1 (2.4%)
1 don’t know	1 (2.4%)
Missing	4 (9.5%)
*Ever Traded Sex for:*	
Food*.*	6 (14.3%)
Money	8 (19.0%)
Drugs	10 (23.8%)
*Ever Diagnosed with STI*	
Yes	26 (61.9%)
Missing	1 (2.4%)
*Ever Diagnosed with:*	
Chlamydia	15 (35.7%)
Gonorrhea	3 (7.1%)
Hepatitis C	1 (2.4%)
Herpes	5 (11.9%)
HIV	2 (4.8%)
HPV	5 (11.9%)
Syphilis	1 (2.4%)
Trichomoniasis	5 (11.9%)

### Contraception decisions and access

Decisions About Contraception Use. Most women reported using birth control to prevent pregnancy (*n* = 33, 79%) and fewer responded that birth control was only used to prevent STIs (*n* = 17, 41%), but 16 (38%) reported they used contraception for both reasons. One participant reported that she used condoms as a contraceptive method because of its protection against both pregnancy and STI:

“I think that’s the safest. From almost, you know, higher chance of preventing a pregnancy and STDs and birth control is just you know getting pregnant but there’s always that chance that you can get something.”

Condom Use (treatment location 1). Participants from treatment location 1 provided insights into reasons for using male condoms. Partner newness was an important factor, but uptake was low. Fewer than 20% said they would use male condoms with a new (*n* = 3) or different (*n* = 3) partner.

“I have only been with one guy since I was 19, which is my kid’s dad. If it was somebody else, I would. Like I used a condom even when we first met but once we got serious we didn’t. So if it was somebody I was seeing casually then yes [a condom would be used].”

Birth control use (treatment location 2). Experience with one’s partner also influenced the decision to use birth control among participants at treatment location 2. Just over 21% of participants (*n* = 4, 21.1%) stated that they would not use birth control with a monogamous partner. Two participants (10.5%) reported they would use birth control with a new partner.

Contraception Access. Only one participant reported ever having difficulty obtaining birth control covered by insurance including Medicaid. Many participants (*n* = 18, 43%) reported birth control prescriptions were obtained through an OB/GYN or healthcare provider. Seven participants (17%) indicated obtaining birth control directly from Planned Parenthood or a clinic.

### Contraceptive preferences and perceptions

Lifetime use of contraception types are reported in Table 4. The majority of participants had used male condoms (69%) and oral contraceptives (60%). Slightly over 40 % reported using hormonal injections. Seventeen percent indicated ever having used a LARC method, with most indicating use of IUDs (12%).

**Table 4:** Contraceptive Methods: Typically Used, Preferred, and Preferred if Freely Provided

		Lifetime Use n (%)	Preferred Method^a^ n (%)	Preferred Method if Freely Provided^a^ n
Contraceptive Method^b^		(^a^42)	(*n* = 35)	(%) (*n* = 37)
*None*				
	None	1 (2.4%)	–	1 (2.7%)
*Tier 1 (Very Effective)*				
	IUD	5 (11.9%)	6 (17.1%)	6 (16.2%)
	Implant	2 (4.8%)	1 (2.9%)	7 (18.9%)
	Tubal Ligation	–	1 (2.9%)	1 (2.7%)
*Tier 2 (Effective)*				
	Oral Contraception	25 (59.5%)	5 (14.3%)	6 (16.2%)
	Nuvaring	3 (7.1%)	–	1 (2.7%)
	Injection	17 (40.5%)	14 (40.0%)	15 (40.5%)
	Hormonal Patch	5 (11.9%)	–	–
*Tier 3 (Moderately Effective)*				
	Male Condom	29 (69.0%)	1 (2.9%)	1 (2.7%)
	Diaphragm	1 (2.4%)		
*Tier 4 Less Effective*				
	Spermicide	2 (4.8%)	1 (2.9%)	–
*Other*				
	Unsure	–	–	2 (4.5%)
	Plan B	5 (11.9%)	1 (2.9%)	–
*Missing*		–	7 (20.0%)	4 (11.0%)

^a^Percentages may total greater than 100% as some participants provided more than preferred or preferred if freely available contraception method

^b^Tier rating based on World Health Organization’s (WHO) four-tiered rating of contraceptive effectiveness

Participant responses regarding preferred and preferred if free contraceptive types are reported in Table 4. When asked about preferred methods of contraception, Tier 1 and Tier 2 methods were preferred. Specifically, 40% of participants indicated a preference for injections, followed by IUDs (17%). Participants were also asked “If you could receive free birth control, would you use it? What type of free birth control would you use and why?” Approximately 90% of participants stated they would use free birth control. Injections were the most frequently reported method participants would use if freely provided, with 41% of women indicating they would choose this type. Implants, IUDs, and oral contraception were the next most frequently reported preferred types of contraception if freely provided.

Eighteen participants indicated a total of 24 reasons why they would use a specific type of contraception if free. Most of these responses centered around not needing to remember to take the contraception (*n* = 10, 56%), side effects with other types of methods (*n* = 5, 28%), and long-term effectiveness (*n* = 4, 10%). Among those who listed not needing to remember to take the contraception, most preferred injections (*n* = 6), followed by the implant (*n* = 3). Of the five women who were concerned about side effects with other types of methods, three preferred LARC methods. For example, one participant cited concerns about problems with oral contraception as the reason she might consider getting an implant:

“The pill was [my preferred method]. . .I hear all these horror stories and something messes up. I wasn’t thinking about getting the thing in my arm. Now maybe I would get the thing in my arm.”

Four participants indicated they preferred their reported choice due to the method’s long-term effectiveness. The methods preferred by these women were LARC. For example, one woman reported her preference for an implant due to its long-term effectiveness. This participant also indicated concerns about problems as a reason for her preference of the implant over the IUD:

“Just cause it lasts. Well, I know the IUD lasts for 5 years and the implant lasts for 3, but I’ve heard, like I know a few people that have a lot of problems with the IUD and I haven’t heard of a lot of issues with the implant.”

Participants at treatment location 2 were also asked about perceived effectiveness of contraceptive methods; participants indicated the percentage or number of women out of 100 who would get pregnant in a year using different methods (Table 5). Tubal ligation, followed by injections and implants were perceived to be the most effective methods with 68%, 40% and 36% of participants, respectively, indicating that less than one woman out of 100 would get pregnant after the procedure. Participants were the least certain about diaphragms, spermicides, the ring, and the patch with regards to the percentage of women who would get pregnant in a year using those methods.

**Table 5:** Frequency of Responses to the Question “What Percentage (or number of women out of 100) do you think would get pregnant in a year using the following methods?” (*n =* 25)

		< 1%	1–5%	6–10%	> 10%	Don’t Know
Contraceptive Method^a^		n (%)	n (%)	n (%)	n (%)	n (%)
*Tier 1 (Very Effective)*						
	IUD	5 (20.0%)	8 (32.0%)	5 (20.0%)	1 (4.0%)	6 (24.0%)
	Implant	8 (36.0%)	4 (16.0%)	4 (16.0%)	–	9 (36.0%)
	Tubal Ligation	17 (68.0%)	5 (20.0%)		1 (4.0%)	2 (8.0%)
*Tier 2 (Effective)*						
	Oral Contraception	1 (4.0%)	7 (28.0%)	10(40.0%)	4 (16.0%)	3 (12.0%)
	Vaginal Ring	3 (12.0%)	6 (24.0%)	5 (20.0%)	–	11 (44.0%)
	Injection	10 (40.0%)	7 (28.0%)	3 (12.0%)	–	5 (20.0%)
	Hormonal Patch	1 (4.0%)	10 (40.0%)	1 (4.0%)	3 (12.0%)	10 (40.0%)
*Tier 3 (Moderately Effective)*						
	Condom (male or female)	–	7 (28.0%)	10 (40.0%)	7 (28.0%)	1 (4.0%)
	Diaphragm	2 (8.0%)	6 (24.0%)	2 (8.0%)	1 (4.0%)	14 (56.0%)
*Tier 4 Less Effective*						
	Spermicide	4 (16.0%)	5 (20.0%)	3 (12.0%)	1 (4.0%)	12 (48.0%)
*None*						
	No method	1 (4.0%)	1 (4.0%)	–	22 (88.0%)	1 (4.0%)

^a^Tier rating based on World Health Organization’s (WHO) 4 tiered rating of contraceptive effectiveness

Participants at location 2 also reported how much they were bothered by the different the dangers/side effects of the different contraceptive methods (Table 6). The most common response was “I don’t know” for IUDs, implants, vaginal rings and diaphragms. For tubal ligation, oral contraception, injection, hormonal patch, condom, family planning, and spermicide the most frequent response was “Not at all” or “A little”.

**Table 6:** Frequency of Responses for the Question “How bothered are you or would you be by the dangers or side effects” (*n* = 25)

		“Not at all” & “A Little”	“A lot” & “Extremely”	“I Don’t Know”
Contraceptive Method^a^		*n* (%)	*n* (%)	*n* (%)
*Tier 1 (Very Effective)*				
	IUD	10 (40.0%)	3 (12.0%)	12 (48.0%)
	Implant	9 (36.0%)	4 (16.0%)	12 (48.0%)
	Tubal Ligation	13 (52.0%)	6 (24.0%)	6 (24.0%)
*Tier 2 (Effective)*				
	Oral Contraception	17 (68.0%)	1 (4.0%)	7 (28.0%)
	Vaginal Ring	10 (40.0%)	3 (12.0%)	12 (48.0%)
	Injection	18 (72.0%)	2 (8.0%)	5 (20.0%)
	Hormonal Patch	13 (52.0%)	1 (4.0%)	11 (44.0%)
*Tier 3 (Moderately Effective)*				
	Condom (male or female)	20 (80.0%)	2 (8.0%)	3 (12.0%)
	Diaphragm	11 (44.0%)	1 (4.0%)	13 (52.0%)
*Tier 4 Less Effective*				
	Spermicide	13 (52.0%)	1 (4.0%)	11 (44.0%)

^a^Tier rating based on World Health Organization’s (WHO) 4 tiered rating of contraceptive effectiveness

## Discussion

The current research confirms and expands upon prior work examining contraception decisions, preferences and perceptions with a sample of current or recently pregnant in-treatment women in the United States who use opioids.

### Decisions to use contraception

Nearly all our participants reported easy access to birth control. Most participants indicated past or present use of Medicaid, which has historically covered family planning services and also mandates recipients be entitled to choose a method of contraception [29]. However, despite reporting easy contraception access, similar to other drug using populations [1], our sample had an unintended pregnancy rate of 75%. Further, only five of the most recent pregnancies were intended, suggesting low contraception use rates and/or use of less effective means of contraception. Correspondingly, Terplan et al. [30] reported that contraceptive use among opioid and other drug-using women ranged from 6% to 74% with the median rate of 55%.

Comparable to research conducted by Olsen et al. [27], participants provided a diversity of situations in which they would or would not use contraception. However, familiarity with one’s current partner was the most cited reason for why a participant would or would not use contraception with nearly a third of participants reporting they would not use contraception with a monogamous partner. Conversely, nearly 30% of participants stated they would use contraception if with someone they did not know very well or with someone other than their monogamous partner. Likewise, prior research has found that, among female teenagers who are intravenous drug users, being engaged in a longer-term sexual relationship significantly decreased the likelihood of using contraception [31].

### Contraception method preferences and perceptions

In our sample, LARC and injections shared comparable perceived benefits, yet injections were much more highly preferred. The opioid-using women in our study demonstrate similar perceptions as non-drug-using women in the United States about LARC contraception methods. This includes concerns and lack of knowledge about side effects, overestimation of efficacy of all forms of Tier 2 and Tier 3 methods, and overreliance on verbal testimonies of other women’s experiences with LARC methods [32–34]. Substance abusing women have been demonstrated to hold additional perceptions that may impact LARC preferences including: misperception of fertility; underestimation of the benefits of LARC; overestimation of the costs and risks associated with LARC; intimate partner violence; fear of losing custody of children; and denial or embarrassment of their substance use [21–23]. Understanding the factors that may make women hesitant about LARC methods relative to injections is important because injections have not been demonstrated to have high rates of use among drug-using women [25] and at least one study has demonstrated reduced injection adherence among an opioid using sample [12]. Sinha et al. reported that of 14 postpartum opioid-using women who received injectable contraception post pregnancy, none continued with injections beyond their initial administration. However, of the 20 women who received implants, 95% continued with this method of contraception [12].

Educational programs for women that accurately explain the side effects, effectiveness and safety associated with Tier 1 LARC products may be an effective way to help opioid-using women decide which product is correct for them and thus increase adherence and decrease unwanted pregnancies. Similar projects have been found to be effective in informing women about their contraceptive decisions [35, 36].

Contraceptive CHOICE provided contraception counseling to over 9200 women and teenagers recruited from abortion clinics in St. Louis, Missouri [37]. Participants were educated about all reversible contraception choices with the benefits of long-acting reversible contraception highlighted. Participants selected the freely provided contraceptive method of their choice. Relative to national levels, the researchers found significantly lower rates of teenage pregnancy and abortions within their cohort. Another study that provided patient education, The Family Planning Initiative, provided approximately three hours of contraception education to 681 pregnant or recently post-partum substance abusing women [36]. Participants were offered freely available contraception of their choice. Participants chose LARC, particularly implants, at slightly relatively higher levels (14% among postpartum women) in comparison to general public utilization [38]. These findings hold promising implications for the benefits of patient education on LARC methods; however, further research is needed to warrant the effectiveness of specific approaches to patient education, including how providers interact with patients when helping them choose a method of contraception [39, 40].

### Limitations and future research

There are several limitations to the current study that merit discussion. First, generalizability is limited due to our relatively small and geographically limited sample. Second, several limitations are related to measurement methods. Because the measures were self-report, the possibility exists for inaccurate or biased recall and/or socially desirable responses. Further, some questions differed between treatment locations preventing the compilation of data across both sites. Additionally, our study would have benefited from greater precision when assessing perceptions of effectiveness and the dangers or side effects of different contraception methods. For example, other research has defined efficacy as 100 minus the “method’s first year failure rate in typical use”. Correspondingly, when estimating efficacy, participants are asked to indicate which of the following choices represent the effectiveness for each method: 99, 95, 91, 83, and 70% [33]. It would have been helpful if our study had qualified efficacy as applying to either perfect or typical use and also used similar response options.

Third, in addition to limited generalizability and measurement weaknesses, collection of additional relevant data would have assisted with the interpretation of results. While we focused on decision making, preferences and perceptions, assessing which women were currently sexually active, planning on a future pregnancy, and/or not currently using contraception would have been useful. Further, despite our finding that 79% of the women used contraception to prevent pregnancy, our survey did not ask the women what priority they assigned to contraception—either its initiation or continuation of use. Consequently, we do not know whether unintended pregnancy was a sufficiently negative event to motivate the use of contraception among our sample. Recent findings indicate that many women do not think a woman should plan for pregnancy [41], and this would be important to assess among opioid-using women. Additionally, while we addressed lifetime utilization of Medicaid, it would have been informative to measure participants’ access to Medicaid during pregnancy-only versus time periods outside of pregnancy. Lastly, women in this study stated that they would use free contraception if it were available. However, this study does not assess actual uptake of specific modes of contraception under those circumstances.

Despite limitations, this study provides additional insight into the contraception decision-making, perceptions and preferences of pregnant and recently pregnant opioid-using women, a complex and understudied population, particularly in the United States. Future research that operationalizes key concepts, such as conditions under which contraception is used, as well as inquires about the acceptability of specific contraceptive methods, is warranted among a larger sample of opioid-dependent women. For example, the current research was conducted in a treatment facility that included both residential and non-residential treatment services. It would be important to understand how contraception choices are impacted by stage of recovery and setting.

Additionally, it is necessary to investigate how or whether healthcare providers are presenting and discussing contraception options with this population. For example, to what extent are the safety and effectiveness of LARC methods such as IUDs and implants being discussed with patients and misconceptions remedied? Likewise, given the high rates of STIs in drug using populations [42, 43], providers may be focused on promoting condom use as a means to prevent STIs, despite only moderate effectiveness in preventing pregnancy.

## Conclusion

Nearly all participants stated they would utilize contraception if freely available, yet high rates of unintended pregnancy were measured in our study population. However, despite participant reported ease of access to contraceptives, and participants reported perceived benefits of LARC, very few had ever used these methods. This indicates the need to address potential patient-level barriers, such as LARC misperceptions, through research on the impact of patient education for both opioid-using and non-opioid using women. Further, we must continue to listen to the voices of women, whose feedback in recent literature has consistently stressed the importance of education on effective methods of contraception and education on the risks and benefits associated with them.

## Abbreviations

IUD: Intrauterine Device
LARC: Long-Acting Reversible Contraception
NAS: Neonatal Abstinence Syndrome
STI: Sexually Transmitted Infections
